# 

**Published:** 1883-02

**Authors:** 


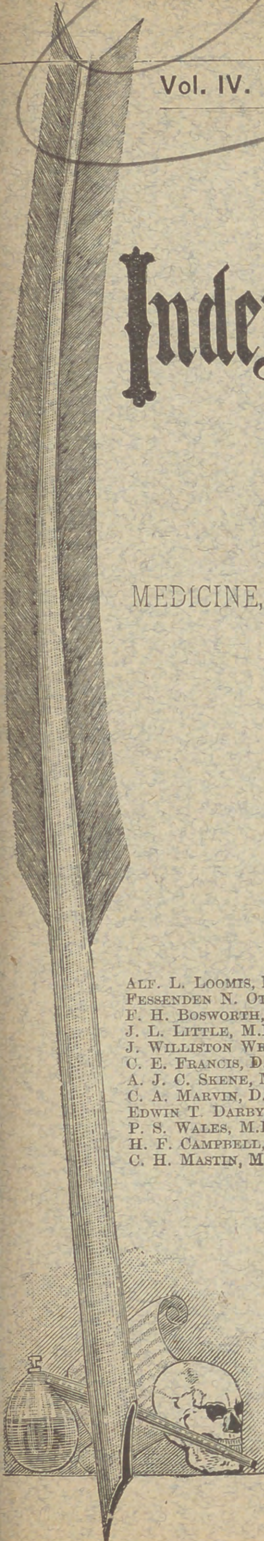

Vol. IV. FEBRUARY, 1883. No. 2.

THE

### Independent Practitioner:

A MONTHLY JOURNAL,

DEVOTED TO

MEDICINE, SURGERY, OBSTETRICS, DENTISTRY, PATHOLOGY AND POPULAR SCIENCE.

EDITORS:

LEIGH H. HUNT, B.Sc., M.D.

W. C. BARRETT, D.D.S., M.D.

NEW YORK:

*THE COLUMBIA PUBLISHING CO.,*

42 DUANE STREET.

**$2.50 Per Annum in Advance. - - Single Copies, 25 Cents.**

Entered at the Post-Office, New York City, as Second-Class matter.

